# Air Pollution and Gerontological Constructs Among Patients With End-Stage Kidney Disease

**DOI:** 10.1093/geroni/igaa057.866

**Published:** 2020-12-16

**Authors:** Mara McAdams-DeMarco, Miranda Jones, Yijing Feng, Jeremy Walston, Nadia Chu, Dorry Segev

**Affiliations:** 1 Johns Hopkins Bloomberg School of Public Health, Baltimore, Maryland, United States; 2 Johns Hopkins School of Medicine, Baltimore, Maryland, United States

## Abstract

Frailty is triggered by inflammatory pathways among patients with end-stage kidney disease (ESKD). Exposure to air pollution is associated with increased inflammation and as such may be a determinant of frailty in patients with ESKD. Therefore, we sought to estimate the impact of household-level exposure to fine particulate matter (particles <2.5μm in diameter [PM2.5]) on frailty and other gerontological constructs among patients with ESKD. We leveraged a prospective, two-center cohort study of 1,482 adults with ESKD (2014-2019) from 40 US states. The physical frailty phenotype (PFP), SPPB, ADL/IADL dependence and 3MS global cognitive impairment were assessed at transplant evaluation. Household-level air pollution was estimated as annual average PM2.5 concentrations at each participant’s address using SEDAC national air pollution data. We estimated the odds of these gerontologic constructs using adjusted logistic regression by quartiles of PM2.5 concentrations accounting for confounders including socioeconomic status. Compared to patients with PM2.5 concentrations in the lowest quartile (<9.3µg/m3), those with exposure to the 3rd quartile (10.0-11.1µg/m3) had 1.50-fold (95%CI:1.04-2.17) increased odds of frailty. However, exposure to PM2.5 concentrations in the second (9.3-10.0µg/m3) and fourth quartiles (>11.1µg/m3) were not significant. Those with PM2.5 in the 3rd (OR=1.60, 95%CI:1.19-2.16) or 4th (OR=1.61, 95%CI:1.20-2.16) quartile had an increased risk of having dependence in ADLs or IADLs. PM2.5 was not associated with SPPB or cognitive impairment. Among ESKD patients, fine particulate matter was associated with greater frailty and dependence burden, although these association may not be linear. Further study of the role of inflammation on these associations are needed.

